# Orthodontic apps: an assessment of quality (using the Mobile App Rating Scale (MARS)) and behaviour change techniques (BCTs)

**DOI:** 10.1186/s40510-021-00373-5

**Published:** 2021-09-13

**Authors:** N. R. Siddiqui, S. J. Hodges, M. O. Sharif

**Affiliations:** 1 Eastman Dental Hospital, University College London Hospitals Foundation Trust, London, UK; 2 Department of Orthodontics, University College London, London, UK

**Keywords:** Apps, Orthodontics, Smart phones, Social media, Behaviour change, Health technology

## Abstract

**Background:**

Apps have been shown to be an effective tool in changing patients’ behaviours in orthodontics and can be used to improve their compliance with treatment. The Behaviour Change Techniques (BCTs) and quality (using MARS) within these apps have previously not been published.

**Objectives:**

1. To evaluate the quality of these apps aiming to change behaviour.
2. To assess BCTs used in patient focused orthodontic apps.

**Methods:**

The UK Google Play and Apple App Stores were searched to identify all orthodontic apps and 305 apps were identified. All 305 apps were assessed for the presence of BCTs using an accepted taxonomy of BCTs (Behaviour Change Wheel (BCW)), widely utilised in healthcare. Of those containing BCTs, the quality was assessed using the Mobile App Rating Scale (MARS), a validated and multi-dimensional tool which rates apps according to 19 objective criteria. Data collection was carried out by two calibrated, independent assessors and repeated after 6 weeks for 25% of the apps by both assessors.

**Results:**

BCTs were found in 31 apps, although only 18 of them were analysed for quality and 13 apps were excluded. Six different BCTs were identified: these were most commonly ‘prompts/cues’, and ‘information about health consequences’. All apps were shown to be of moderate quality (range 3.1–3.7/5). Inter-rater and intra-rater reliability for BCT and quality assessment were excellent.

**Conclusions:**

The current availability of orthodontic apps of sufficient quality to recommend to patients is very limited. There is therefore a need for high-quality orthodontic apps with appropriate BCTs to be created, which may be utilised to improve patients’ compliance with treatment.

## Background

Successful orthodontic outcomes rely on excellent patient compliance. Non-compliance can lead to compromised treatment results and an economic burden to the patient and clinician. Mandall et al. showed that 43% of orthodontic patients did not complete treatment, mainly due to poor compliance, such as multiple failed appointments (43%), poor oral hygiene (31%) and appliance breakages (16%) [1]. Risks associated with non-compliance include demineralisation, caries, periodontal disease, trauma and root resorption [2].

Improving compliance necessitates a change or modification of behaviours and traditionally, in dentistry, information provision (e.g. verbal, written, videos, social media) has been used most commonly in an attempt to achieve this. However, traditional methods of providing information alone may be insufficient to improve compliance due to the limitations in a number of these. This may be due to the fact that behaviour change is dependent on a set of key factors including cognition, attitude and cultural variation [3]. To this end, a systematic review concluded that there was insufficient evidence to allow orthodontists to choose a single method to improve compliance in our patients [4].

The Behaviour Change Wheel (BCW ) is a comprehensive, evidence-based and widely accepted model used to develop effective behaviour change [5]. It has been used by the UK’s National Institute for Health and Care Excellence’s guidance on reducing obesity and the Department of Health’s 2010 tobacco control strategy. The incorporation of behaviour change theory into interventions is likely to increase their effectiveness. Behaviour Change Techniques (BCTs) are activities designed to change behaviour patterns, including changes aiming to improve compliance, and they aim to address components of the BCW. Ninety-three BCTs have been recognised, with some examples relevant to orthodontics which could be incorporated into apps detailed in Table 1.

**Table 1:** Behaviour change technique themes with examples related to orthodontics

Theme	Example(s)
Feedback and monitoring	Patient completes a daily chart of how many hours they have worn headgear, and this can be self-monitored or monitored by the clinician with feedback.
Shaping knowledge	Show the patient a video on how to insert, remove and look after an appliance.
Repetition and substitution	Practice placing elastics with the patient in the dental chair to ensure they are confident to do it alone at home.

Smartphones are accessible/portable, interactive and can provide a diverse range of features and therefore may address some of the previous challenges to instigate behaviour change. Recently, the availability of apps has provided an alternative method to attempt to change behaviour and improve compliance. There is a rapidly increasing availability of apps, with 305 orthodontic apps being identified in the UK Apple App Store and Google Play Store in 2019 [6–8]; additionally, there is a demand by patients to use apps to aid with treatment [9]. It is possible that there will be better engagement with apps than other methods of information provision. A number of randomised controlled trials have shown apps to be an effective tool to improve outcomes in orthodontic patients, including improving plaque indices, reducing the presence of white spot lesions, reducing treatment time and fewer failed appointments [10–13].

However, the rapid proliferation of apps makes it increasingly difficult for patients and professionals to identify high-quality apps. To date, there has been no formal published assessment on the quality of orthodontic apps. Research on the quality of orthodontic information delivered via social media shows that it is poor, and this has been reported to be due to the fact that the majority of information was uploaded by patients rather than professionals, and this may be similar for apps [14, 15]. The information provided within apps may be used to help patients make informed decisions about their healthcare, and the quality of this information should therefore be accurate, evidence-based and peer-reviewed; however, there are currently no regulatory standards for the content published within healthcare apps. The app store users rate apps using a 5-point Likert scale; however, selecting apps on the basis of user popularity does not necessarily correlate to the quality of the app [16]. Chen et al. evaluated the quality of the 800 most popular health and fitness smartphone apps and showed that the most popular apps scored below average on a number of quality features [17]. The NHS library of apps lists those apps which meet NHS quality standards for safety and user-friendliness; however, no orthodontic apps are available in this library [18].

A number of assessment tools have previously attempted to analyse the quality of healthcare apps but were found to be too general, specific or complex [19–21]. This led to the development of the Mobile App Rating Scale (MARS), which is an evidence-based, objective, multidimensional measure for rating the quality of mobile health apps [22]. It is reported to be comprehensive, yet easy to use with minimal training allowing routine use in practice and research. The MARS addresses four domains which are summarised in Table 2.

**Table 2:** Mobile App Rating Scale categories with descriptions

Category	Description
Engagement	Evaluates entertainment, interest, customisation, interactivity and target group.
Functionality	Evaluates performance, ease of use, navigation and gestural design.
Aesthetics	Evaluates layout, graphics and visual appeal, i.e. how good does the app look?
Information	Evaluates accuracy of the app description, app goals, quality and quantity of information, visual information, credibility and evidence base.

These four domains are subdivided into 19 individual criteria, each of which is scored against a 5-point Likert scale (maximum score = 95), from which an overall mean app quality score can be calculated (out of five). The higher the mean score, the higher the quality of the app. Mean scores are also calculated for each of the four domains to highlight areas of the apps strengths and weaknesses.

There has been a rapid proliferation of apps and the quality of orthodontic apps in the UK previously has not been comprehensively assessed and published. This information may allow us to identify high-quality apps, aimed at improving patient compliance.

## Objectives

1. To evaluate the quality of patient focused orthodontic apps aiming to change behaviour using MARS.
2. To assess BCTs used in patient focused orthodontic apps using an accepted taxonomy (BCW).

## Design

Cross-sectional review study.

## Setting

All available orthodontic apps on the UK Google Play and Apple App Stores.

## Methods

This study was a review of apps conducted in London, UK in 2020. Data collection was in two stages:

- Stage 1: Assessment of BCTs used in patient focused apps aiming to elicit a behaviour change.
- Stage 2: Quality assessment of the apps identified in stage 1, using MARS.

### Selection criteria

In a preliminary study, the authors carried out a review of all available orthodontic apps on the UK Google Play and Apple App Stores [23]. From the 305 apps identified, the following apps were included:

- Patient focused apps aiming to elicit a behaviour change.
  - An app aiming to elicit a behaviour change was defined as an app which used at least one of the 93 BCTs defined by the BCW Taxonomy [5].
- Free or paid apps.
- Accessible apps (e.g. functional, no password required)

### Training and calibration

All assessors undertook BCT and MARS training devised by the authors of the techniques prior to app assessment. Behaviour Change Technique training was devised by the founders of the BCW and consisted of an online training programme including assessments. It consisted of a number of excerpts from various healthcare journals, in which the BCT(s) had to be identified and scored for their strength. One of the assessors (MOS) also attended a 5-day course at the Centre for Behaviour Change, University College London in July 2017. Mobile App Rating Scale training consisted of a 92-slide power point presentation and a 37-min YouTube video.

The assessors were also calibrated in these two techniques, as per the authors’ advice [22]. Raters practiced scoring on the app ‘Happier’ and completed a self-assessment exercise. The assessors compared and discussed their results and, following this, one app was selected at random to calibrate the MARS and BCT scoring of the two assessors. Following this, a further three apps were assessed independently by the assessors and compared. This was followed by rounds of assessment (three apps at a time) until a shared understanding of app rating was achieved by the assessors and an adequate level of inter-rater reliability was achieved. It was intended that if there was any disagreement between assessors then these would be discussed to obtain consensus. Where consensus was not obtained, a third assessor (SJH) would be consulted to mediate and obtain consensus.

### Data collection

Microsoft Excel was used to record at least one of the 93 BCTs for each included app and for the MARS assessment each of the 19 app features was scored on a 5-point Likert scale. A rating of 3 was used as a baseline if the feature was ‘average’. A score of 1 or 2 was given if necessary components had not been included. A score of 4 or 5 suggested that the feature was exceptional or innovative. The total objective score of the 19 features was calculated, as was the mean score.

### Inter-rater and intra-rater reliability

Data collection for all apps was carried out by two assessors (NRS and MOS). Where there was any disagreement between assessors for BCT assessment, agreement was reached by consensus, and a third assessor (SJH) was to be consulted if agreement could not be reached. Where there was any disagreement for MARS assessment, a mean score was calculated. Data collection was repeated by the two independent assessors for 25% of the apps 6 weeks following initial assessment. The selection of apps was chosen using a random number generator.

## Results

### Stage 1—BCT assessment

Thirty-one apps aimed to change behaviour, of which 13 were excluded as they required a login or were non-functional, and therefore 18 apps were analysed. All 18 apps were free of charge. There was 100% inter-rater and intra-rater reliability. Six out of a possible 93 BCTs were identified within these apps, most commonly prompts and cues. The prevalence of each BCT can be seen in Table 3.

**Table 3:** Summary of the BCTs identified with their prevalence

BCT	Number of apps
Prompts and cues	17
Information about health consequences	12
Self-monitoring of outcomes of behaviour	6
Instruction on how to perform a behaviour	4
Self-monitoring of behaviour	2
Social reward	1

### Stage 2—quality assessment (MARS)

The overall mean MARS score amongst the 18 apps was 3.4/5 (range 3.1–3.7/5). Of the four MARS domains, functionality scored the highest (3.9/5), followed by aesthetics (3.5/5), information (3.3/5), and engagement (3.0/5). Inter-rater reliability was 89% and intra-rater reliability was 100%.

## Discussion

Previous orthodontic app analysis has been restricted, mostly assessing the focus of available apps [6–8]. This was the first study to assess BCTs and the quality of orthodontic apps using validated tools (BCW and MARS). App assessment was completed independently and in duplicate to strengthen the robustness of the findings. The main limitation of this study was that only 18 of the 31 apps aiming to change behaviour were accessible due to apps being removed from the App stores; apps were non-functional or requiring password protected logins.

### Behaviour change techniques in orthodontic apps

#### Prompts/cues (*n* = 17)

A prompt is a reminder to bring about an event and according to the authors of the BCW taxonomy [5]; they are one of the most frequently used BCTs. They are popular because they are a simple, effective and easily executed feature via an app and benefit patients by reminding them to carry out desired behaviour. The prompts/cues found within the apps assessed included reminders for appointments, elastics, aligners, removable appliances, rapid maxillary expansion, headgear and retainers. The degree of customisation varied significantly between apps, for example, some apps asked a number of questions including how often aligners are to be changed, the time of day to be alerted and start date, whereas other apps only asked how often aligners are to be changed and the patient could not choose the time of day which they receive this prompt.

#### Providing information about health consequences (*n* = 12)

The consequence(s) of non-compliance was discussed in most apps in an attempt to deter the patient against the undesired behaviour. Most apps focussed on the risks associated with poor diet or poor oral hygiene.

#### Self-monitoring of outcomes of behaviour (*n* =6)

All apps containing this BCT allowed a video to be developed made up of selfies showing treatment progress to monitor the outcome of treatment. The aim of this BCT is to reinforce positive behaviour.

#### Instruction on how to perform a behaviour (*n* = 4)

In 3 of the 4 apps containing this BCT, instructions on tooth brushing were given with computer-generated toothbrushes on a virtual 3D model of teeth. One app allowed customisable instructions on elastic wear (the clinician or patient could draw onto a set of teeth the desired configuration of the elastics).

#### Self-monitoring of behaviour (*n* = 2)

This allows the user to monitor their behaviour by recording how often they comply with treatment, e.g. headgear wear, elastic wear and oral hygiene scores. This is different from ‘self-monitoring outcomes of behaviour’ in that the latter measures the outcome of the behaviour rather than the frequency of the behaviour itself.

#### Social incentives (*n* = 1)

This BCT was used in one app to reward the user for complying with the oral hygiene and headgear regimes by way of a points scale.

### Comparison of BCTs in orthodontic apps to BCTs in other healthcare apps

As BCT assessment in orthodontic apps has not been previously reported, the BCTs identified were compared to the wider literature. A systematic review of BCTs in apps across healthcare showed that amongst 64 health apps, there were a limited number of BCTs present, this correlates to the findings of this present study [24–30]. However, the most popular BCTs within this study differed to the ones found within the wider healthcare literature because many of the wider healthcare studies assessed the presence of BCTs targeted to preventing adverse behaviour (e.g. smoking cessation) rather than encouraging positive behaviour (e.g. a prompt to wear an appliance).

### Areas for future development regarding BCTs in orthodontic apps

A systematic review and meta-analysis based on 85 studies across healthcare suggested that the greater the use of behaviour change theory and the greater number of BCTs delivered in an intervention, the greater the effect size of the planned behaviour being carried out (p = 0.049 and p < 0.001 respectively) [31].

Incorporating more BCTs into apps may increase the chances of changing behaviour, for example, extending the BCT ‘instruction on how to perform a behaviour’ into ‘demonstration of the behaviour’ which requires an observable sample of the performance of the behaviour rather than just providing the information. This could be executed via the use of a video being uploaded onto an app demonstrating the desired behaviour. It may be costly to construct high-quality videos; however, undoubtedly useful, and may be more realistically incorporated into a paid app. However, not all of the 93 BCTs would lend themselves to being available via an app.

### Quality assessment using MARS

The mean quality of all assessed apps was average (range 3.1–3.7/5) with functionality being the highest scoring domain (mean 3.9/5), followed by aesthetics (mean 3.5/5). Interestingly, information (mean 3.3/5) and engagement (mean 3.0/5) were the lowest scoring domains.

Information includes assessment of quality, quantity, credibility of the source and evidence basis of information. The quality of information was sometimes inaccurate and potentially dangerous, e.g. advising patients to carry out emergency orthodontic treatment at home, including cutting long arch wires with nail clippers. If an app scores highly in the information domain, it does not mean that all of the content is clinically safe, as this can be masked by high scores in other areas. In addition, none of the assessed apps were developed by a notable credible source or professional body, although a number were developed by specialist practices, or had undergone robust scientific testing which would have improved the scores in the information domain.

*The* three highest scoring apps scored 3.7, 3.6 and 3.6/5, respectively. These apps consistently scored well on three domains (functionality, aesthetic and information) and lowest on engagement. These apps were simple, visually appealing and contained useful features including a calendar recording aligner wear, a number of customisable reminders (for removable appliances, aligners, elastics and rapid palatal expanders) and an excellent educational quiz on topics such as oral hygiene and appliance maintenance with ‘true’ or ‘false’ answers.

The two lowest scoring apps (both scoring 3.1/5) were both designed by specialist practices and were specifically aimed at their own cohort of patients. As described using the terminology in the MARS framework, both apps were deemed to be ‘visually boring’, ‘stylistically inconsistent’ and provided a number of features which were not generalisable to patients who were not treated by these specialist practices.

### Comparison of MARS scores in orthodontic apps to MARS scores in other healthcare apps

Since there are no previous published reports of quality assessment in orthodontic apps, the authors compared the MARS results to other studies within healthcare. A literature search showed that these findings are consistent with healthcare apps in general where most are deemed to be low to average in quality with very few apps scoring above 4.0/5. Additionally, functionality consistently scored the highest, engagement consistently scored the lowest and improvement was suggested across all apps [32–35].

### Areas for future development regarding quality improvement in orthodontic apps

The MARS scores showed that the main areas requiring improvement were ‘engagement’ and ‘information’. Improving the information content is of utmost priority in future research as a disengaging app is not dangerous; however, providing incorrect information potentially is as patients may be using the information to inform treatment decisions. There is therefore a need for app information content to be as accurate and evidence-based and assessed by clinicians for accuracy prior recommending them to patients.

Engagement relates to the design and interest of the app and software functionality making the app entertaining with targeted and interactive features. This domain could be improved in future apps through the use of videos, pictures and customisable features which is an achievable goal and the app should be regularly updated to stimulate repeated use, rather than a stale set up which has been found in all of the apps in this study.

### Implications for practice and future research

The current availability of apps provides us with a very limited choice from which to recommend an app for patients; however, the availability and content of the apps is constantly evolving. Therefore, it is important for clinicians to keep abreast of available apps and be able to critically appraise them. The authors of this study feel the most important features for a clinician to look for in a new app, which they may be considering recommending to patients are quality of information, ease of use, entertainment and visual appeal.

Future research into providing high-quality, effective apps, which aim to improve patient compliance, may have numerous benefits in orthodontics including improving treatment outcomes, reducing risks and costs and thereby benefitting the patient, clinician and health service/service provider. Future research is also needed to assess patient-focused apps in regard to the accuracy of information content. There is also scope to improve the overall quality of orthodontic apps and increase the number of BCTs in apps followed by scientific testing for their effectiveness. It is important that both professionals and patients are involved in app developments.

## Conclusions

The purpose of this study was to identify apps which we might consider recommending to our patients to improve their compliance with treatment; however, the current availability provides us with a very limited choice from which we could consider recommending at the moment. There is therefore a need for high-quality orthodontic apps with appropriate BCTs to be created, which may be utilised to improve patients’ compliance with treatment.
